# Inhibiting NLRP3 inflammasome signaling pathway promotes neurological recovery following hypoxic-ischemic brain damage by increasing p97-mediated surface GluA1-containing AMPA receptors

**DOI:** 10.1186/s12967-023-04452-5

**Published:** 2023-08-24

**Authors:** Yuxin Chen, Xiaohuan Li, Qian Xiong, Yehong Du, Man Luo, Lilin Yi, Yayan Pang, Xiuyu Shi, Yu Tian Wang, Zhifang Dong

**Affiliations:** 1 Growth, Development, and Mental Health of Children and Adolescence Center, Pediatric Research Institute, Ministry of Education Key Laboratory of Child Development and Disorders, National Clinical Research Center for Child Health and Disorders, China International Science and Technology Cooperation Base of Child Development and Critical Disorders, Chongqing Key Laboratory of Translational Medical Research in Cognitive Development and Learning and Memory Disorders, Children’s Hospital of Chongqing Medical University, Chongqing, 400014 China; 2 Department of Medicine, Brain Research Centre, Vancouver Coastal Health Research Institute, University of British Columbia, Vancouver, BC V6T 2B5 Canada

**Keywords:** Hypoxic-ischemic brain damage, NLRP3, Caspase-1, GluA1, p97

## Abstract

**Background:**

The nucleotide-binding oligomeric domain (NOD)-like receptor protein 3 (NLRP3) inflammasome is believed to be a key mediator of neuroinflammation and subsequent secondary brain injury induced by ischemic stroke. However, the role and underlying mechanism of the NLRP3 inflammasome in neonates with hypoxic-ischemic encephalopathy (HIE) are still unclear.

**Methods:**

The protein expressions of the NLRP3 inflammasome including NLRP3, cysteinyl aspartate specific proteinase-1 (caspase-1) and interleukin-1β (IL-1β), the α-amino-3-hydroxy-5-methyl-4-isoxazole-propionicacid receptor (AMPAR) subunit, and the ATPase valosin-containing protein (VCP/p97), were determined by Western blotting. The interaction between p97 and AMPA glutamate receptor 1 (GluA1) was determined by co-immunoprecipitation. The histopathological level of hypoxic-ischemic brain damage (HIBD) was determined by triphenyltetrazolium chloride (TTC) staining. Polymerase chain reaction (PCR) and Western blotting were used to confirm the genotype of the knockout mice. Motor functions, including myodynamia and coordination, were evaluated by using grasping and rotarod tests. Hippocampus-dependent spatial cognitive function was measured by using the Morris-water maze (MWM).

**Results:**

We reported that the NLRP3 inflammasome signaling pathway, such as NLRP3, caspase-1 and IL-1β, was activated in rats with HIBD and oxygen-glucose deprivation (OGD)-treated cultured primary neurons. Further studies showed that the protein level of the AMPAR GluA1 subunit on the hippocampal postsynaptic membrane was significantly decreased in rats with HIBD, and it could be restored to control levels after treatment with the specific caspase-1 inhibitor AC-YVAD-CMK. Similarly, in vitro studies showed that OGD reduced GluA1 protein levels on the plasma membrane in cultured primary neurons, whereas AC-YVAD-CMK treatment restored this reduction. Importantly, we showed that OGD treatment obviously enhanced the interaction between p97 and GluA1, while AC-YVAD-CMK treatment promoted the dissociation of p97 from the GluA1 complex and consequently facilitated the localization of GluA1 on the plasma membrane of cultured primary neurons. Finally, we reported that the deficits in motor function, learning and memory in animals with HIBD, were ameliorated by pharmacological intervention or genetic ablation of caspase-1.

**Conclusion:**

Inhibiting the NLRP3 inflammasome signaling pathway promotes neurological recovery in animals with HIBD by increasing p97-mediated surface GluA1 expression, thereby providing new insight into HIE therapy.

**Supplementary Information:**

The online version contains supplementary material available at 10.1186/s12967-023-04452-5.

## Background

Hypoxic-ischemic encephalopathy (HIE) is the most prevalent type of asphyxia-induced neonatal brain damage during the perinatal period [1]. The incidence of HIE ranges from 1 to 3 per 1000 live births in developed countries and approximately 20 per 1000 live births in developing countries [1–3]. Nearly 25% of surviving children with HIE experience neurodevelopmental delays, neurological dysfunction and learning and memory impairments. Due to serious neurological sequelae, HIE can have a significant negative impact on surviving children and their families as well as society [4, 5]. Hypothermia, hyperbaric oxygen and systemic supportive care are the most common treatments for HIE, but their effectiveness is limited [6, 7]. Therefore, exploring the pathogenesis of HIE and searching for feasible interventions to prevent hypoxia-ischemia (HI)-induced brain dysfunction have become urgent needs.

Innate immunity is the first barrier of the immune system, plays a critical role in defending against and eliminating pathogens, and guides the body to mount an adaptive immune response [8]. Pathogen-associated molecular patterns (PAMPs) are molecular structures encoded by pathogens that are shared among multiple related microorganisms. The function of innate immunity involves recognizing PAMPs through pattern recognition receptors (PRRs) [8, 9]. Inflammasomes, which are important components of innate immunity, are multiprotein complexes formed by PRRs. Among the NOD-like receptor family, NLRP3 is the most extensively studied receptor [9–11]. NLRP3 oligomerizes through the NACHT domain in response to diverse stimuli such as pathogens and stress, recruits apoptosis-related specific protein (ASC) through the pyrin domain, and leads to caspase-1 activation [12, 13]. Activated caspase-1 promotes the maturation of IL-1β and interleukin-18 (IL-18), further recruiting more inflammatory cells and amplifying the inflammatory response [14, 15]. Growing evidence has demonstrated that the NLRP3 inflammasome signaling pathway is significantly activated in brain injuries caused by HI [16, 17]. For example, HI promotes the expression of NLRP3, caspase-1, and IL-1β in patient or animal brain samples, as well as in vitro [18–20]. Neuronal death and behavioral deficits can be prevented by suppressing the NLRP3 inflammasome pathway during stroke [18, 19]. However, whether the NLRP3 inflammasome signaling pathway is activated and its role in HI-induced brain dysfunction in the immature brain remains largely unknown.

As the basic structure of the nervous system, synapses play key roles in transmitting information via the release of neurotransmitters from the presynapse to the postsynapse [21]. Synapses can be classified as excitatory and inhibitory based on the binding effects of neurotransmitters. Glutamate is an excitatory neurotransmitter that binds to excitatory receptors such as *N*-methyl-d-aspartate receptor (NMDAR), AMPAR, and kainate receptor (KAR) [22, 23]. Glutaminergic excitotoxicity is primarily mediated by NMDARs and AMPARs and plays a critical role in HI-induced cell death [3, 24, 25]. HI Sensitivity varies among neurons in the affected region. Irreversible neuronal necrosis occurs in the central part of the ischemia, and neurons around the central part exhibit reversible delayed neuronal death, indicating the potential for intervening in hypoxic-ischemic brain damage (HIBD) [26]. Glutaminergic excitotoxicity is characterized by elevated glutamate release and binding to postsynaptic NMDARs, resulting in excessive Ca^2+^ and H_2_O influx, as well as cytotoxic edema and acute cell dissolution [3, 27, 28]. Numerous studies have explored the mechanisms underlying NMDARs in animals with HIBD and found that NMDAR antagonists can successfully prevent HI-induced neuronal damage [29–31]. However, NMDAR antagonists have a limited therapeutic window, and they impair normal NMDAR-mediated brain processes, such as neural circuit maturation, neuronal survival, and learning and memory [32–34]. Therefore, NMDAR antagonists are not suitable for clinical use in human HIE patients.

Compared to the function and underlying mechanisms of NMDARs, the role of AMPARs in HI-induced brain damage remains largely unclear. Some reports suggest that elevated levels of glutamate can induce the phosphorylation of GluA2 subunits, leading to the endocytosis of GluA2-containing AMPARs. The absence of GluA2 subunits causes excessive influx of Ca^2+^ and subsequent cell excitotoxicity and death [35, 36]. Therefore, the regulation of surface AMPARs may also have the potential to prevent cell death after HI. Indeed, a recent report shows that inhibiting caspase-1 modulates surface AMPAR expression and alleviates chronic social defeat stress, indicating that NLRP3 inflammasome signaling pathway blockers could be useful in the treatment of AMPAR-associated ischemic brain injury [37]. However, the specific mechanism by which the NLRP3 inflammasome pathway affects surface AMPARs remains largely unknown.

In this study, we hypothesized that HI reduces surface AMPARs by activating the NLRP3 inflammasome pathway, thereby causing neural damage. To investigate this hypothesis, we performed a series of in vivo and in vitro biochemical and molecular experiments under ischemia-like conditions. Additionally, multiple behavioral experiments were conducted in well-characterized HIBD models to examine the protective effects by inhibiting the NLRP3 inflammasome pathway on neurological function.

## Materials and methods

### Animals

Sprague-Dawley (SD) rats and wild type (WT) mice were purchased from Army Medical University (Chongqing, China). NLRP3^−/−^ and caspase-1^−/−^ mice were kindly provided by Professor Bo Peng (Fudan University, Shanghai, China). Genomic DNA was extracted from the tail tips and subjected to PCR to confirm the genotype. For NLRP3, common primers (5′-TTCCATTACAGTCACTCCAGATGT-3′), WT primers (5′-TCAGTTTCCTTGGCTACCAGA-3′) and mutant primers (5′-TGCCTGCTCTTTACTGAAGG-3′) were used for genotyping (producing a 666 bp band in WT mice and an 850 bp band in mutant mice). For caspase-1, common primers (5′-ATGGCACACCACAGATATCGG-3′), WT primers (5′-GAGACATATAAGGGAGAAGGG-3′) and mutant primers (5′-TGCTAAAGCGCATGCTCCAGACTG-3′) were used for genotyping (producing a 500 bp band in WT and a 300 bp band in mutant). The knockout efficiency of caspase-1 and NLRP3 was further verified by Western blotting (Additional file 1: Fig. S1).

The rat HIBD model was established as described previously with some modifications [38–40]. In brief, unsexed 7-day-old rats were randomly divided into 4 groups: sham, sham + vehicle, HIBD + vehicle, and HIBD + AC-YVAD-CMK. After the rats were anesthetized with isoflurane, the left common carotid artery was isolated and ligated, and a gelatin sponge was used to promote hemostasis. The rats with HIBD were allowed to recover for 2 h in their nests. Then they were placed in a hypoxic environment (8% O_2_ + 92% N_2_) at 37 °C for 2.5 h. In sham rats, the left common carotid arteries were only isolated but not ligated. After observing normal vital signs, the rats were returned to their nests.

The mouse HIBD model was established as previously described with some modifications [41]. In brief, unsexed 10-day-old mice were randomly divided into 4 groups: WT-sham, WT-HIBD, NLRP3^−/−^-sham or caspase-1^−/−^-sham, and NLRP3^−/−^-HIBD or caspase-1^−/−^-HIBD. After the mice were anesthetized with isoflurane, the left common carotid artery was isolated and ligated, and a gelatin sponge was used to promote hemostasis. The mice with HIBD were returned to their nests for 2 h. Due to the physiological and size differences between rats and mice, the mice were subjected to hypoxic conditions (8% O_2_ + 92% N_2_) at 37 °C for 50 min. In sham mice, the left common carotid arteries were only isolated but not ligated. After observing normal vital signs, all the mice were returned to their nests.

All animals were raised in the Animal Care Center of Children’s Hospital of Chongqing Medical University, where they had unlimited access to food and water. The room was controlled on a 12-h light and dark cycle (8:00–20:00) and at a temperature of 21 °C. The use of animals and their suffering are kept to a minimum. Mortality due to surgery was approximately 2% among SD rats and WT mice and approximately 3% among NLRP3^−/−^ and caspase-1^−/−^ mice. The experiments were performed in a double-blind manner to ensure unbiased results.

### TTC staining

Three days after HIBD surgery, rat brain tissues were collected for TTC staining. After being frozen at − 20 °C for 20 min, the brain was sectioned into 5 coronal slices: middle forebrain and optic chiasm, optic chiasm, funnel stalk, middle of the funnel stalk, and caudal pole of the posterior lobe. The sections were washed and stained with phosphate-buffered saline (PBS) and 2% TTC solution. During the staining process, the sections were flipped every 5 min.

### Antibodies and reagents

Anti-NLRP3 (#ab91413), anti-caspase-1 (#ab1872), anti-IL-1β(#ab9722), anti-GluA1 (#ab31232), anti-GluA2 (#ab133477), anti-sodium potassium ATPase (#ab76020) and anti-VCP/p97 (#ab11433) antibodies were purchased from Abcam. Anti-β-actin antibody (#A5441) was obtained from Sigma-Aldrich. Anti-PSD-95 antibody (#MAB1598) was obtained from Millipore.

The Minute™ Plasma Membrane Protein Isolation and Cell Fractionation Kit (#SM-005) was purchased from Invent Biotechnologies, Inc. Roche Applied Science provided Complete Protease Inhibitor Cocktail Tablets (#04693116001). The Pierce™ BCA Protein Assay Kit (#23225) was purchased from Thermo Scientific. Protein A/G Magnetic Beads (#B23202) was purchased from Bimake. AC-YVAD-CMK peptide (#SML0429) was purchased from Sigma-Aldrich. For the in vitro experiments, cultured primary neurons were pretreated with AC-YVAD-CMK (5 µM) 1 h before oxygen-glucose deprivation (OGD). For the in vivo experiments, AC-YVAD-CMK (1 mg/kg, i.p.) was administered 1 h before HIBD surgery and then daily for 7 days.

### Primary culture of neurons

Neurons were isolated from the brains of D18 fetuses as previously reported with modifications [39, 42]. The fetuses were sterilized in ice-cold alcohol from pregnant SD rats that were anesthetized with urethane (1.5 g/kg, i.p.). The brain tissues were separated with Hank’s balanced salt solution (HBSS) buffer, digested with 0.25% trypsin-ethylene diamine tetraacetic acid (EDTA) for 10 min at 37 °C, washed with Dulbecco’s modified Eagle’s medium (DMEM), filtered with a cell strainer (100 μm), and centrifuged at 600 rpm for 3 min. The precipitate was resuspended in DMEM (containing 10% fetal bovine serum [FBS]) before being plated on dishes coated with poly-d-lysine (PDL) (1.0 × 10^7^ per 10 cm dish, 6.0 × 10^6^ per 6 cm dishes, 2.0 × 10^6^ per 6 well plate). The neurons were incubated (Thermo Forma 3111, Thermo Scientific) with 5% CO_2_ at 37 °C. After 4–24 h, the neurobasal feeding buffer (containing 500 ml Neurobasal medium, 2% SM1 supplement, and 0.5mM GlutaMAX^TM^-I supplement) was used to replace the DMEM buffer (containing 10% FBS). Subsequently, half of the neurobasal feeding buffer was replaced every 3 days.

### OGD

To mimic immature cerebral ischemia in vitro, cortical/hippocampal neurons were exposed to OGD after 7 days of culture as previously described [39]. Neurobasal feeding buffer was replaced with 199/Earle’s balanced salt solution (EBSS). Then, the neurons were placed in a Thermo Scientific incubator for 1.5 h under hypoxic conditions (5% O_2_ + 95% N_2_). Then the 199/EBSS buffer was replaced with the previously saved neurobasal feeding buffer. The neurons were allowed to recover for 0 to 24 h in incubator with 5% CO_2_ at 37 °C.

### Western blotting

Total proteins were extracted in ice-cold NP-40 buffer (Beyotime, China) containing a protease inhibitor. Synaptic proteins were extracted as previously described [43, 44], and membrane proteins were extracted by a Minute™ Plasma Membrane Protein Isolation and Cell Fractionation Kit. The protein concentration was measured by a Pierce™ BCA Protein Assay Kit. The aliquoted proteins were boiled with 5× sample buffer for 5 min at 95 °C.

The samples of protein (30 µg and 10 µg for total and membrane or synaptic protein, respectively) were separated by sodium dodecyl sulfate (SDS)-polyacrylamide gel electrophoresis (PAGE) gels (10–15%). Then, the protein samples were transferred to polyvinylidene difluoride (PVDF) membranes at 105 volts for 105 min at 4 °C. The membranes were incubated with 5% fat-free milk for 2 h at room temperature. Anti-NLRP3 (1:1000), anti-caspase-1 (1:500), anti-IL-1β (1:1000), anti-GluA1 (1:500), anti-GluA2 (1:1000), and anti-VCP/p97 (1:1000) were added and incubated with the membranes overnight at 4 °C. The membranes were then incubated with the corresponding secondary antibody (1:3000) or fluorogenic secondary antibody (1:10,000) at room temperature for 30–90 min, imaged with the Bio-Rad Imager or ODYSSEY Infrared Imager System (LI-COR, Inc.) and quantified with Bio-Rad Quantity One software. β-actin (1:3000), PSD-95 (1:500) and NaK-ATPase (1:1000) served as loading controls for total proteins, synaptic proteins and membrane proteins.

### Co-immunoprecipitation (Co-IP)

Cultured primary neurons were lysed in IP lysis buffer (containing a protease inhibitor). Protein A/G Magnetic Beads were used to separate the cell debris and the concentration was measured by a Pierce™ BCA Protein Assay Kit. The lysates were aliquoted into two parts: in the input groups, the proteins, IP lysis buffer and 5× sample buffer were mixed, and in the IP groups, the proteins, IP lysis buffer and antibodies were mixed. The IP groups were incubated with anti-GluA1 or anti-VCP/p97 overnight at 4 °C. After the magnetic beads and the IP group samples were mixed for 2 h at 4 °C, IP lysis buffer and 5× sample buffer were added to the magnetic beads, and the target proteins were boiled for 5 min at 95 °C.

### Grasping test

After 3 weeks of recovery from HIBD surgery, the rats/mice were subjected to the grasping test. The grip of the forelimbs was tested by a Grip Strength Meter (Chatillon, USA). The animals were evaluated 5 times (10-min intervals). Myodynamia was calculated using the mean values.

### Rotarod test

Twenty-four hours after the grasping test, the rats/mice were subjected to the rotarod test. The rats/mice were pretrained for 2 rounds at constant speeds of 10 rpm and 20 rpm (3 min for rats, 5 min for mice). The rats were trained for 10 rounds at a constant speed from 10 to 55 rpm (5 rpm increases, 20-min intervals). The mice were trained for 8 rounds at the constant speed from 5 to 40 rpm (5 rpm increases, 20-min intervals).

### Morris water maze (MWM) test

Four weeks after HIBD surgery, the MWM test was performed to evaluate spatial cognitive function as described previously [39, 44]. Prior to spatial training, nontoxic paint was used to darken the pool water (25 ± 1 °C). The animals were permitted to swim freely for 60 or 120 s to adapt to the testing conditions. The spatial learning task was performed in the next 5 days. During spatial learning, a platform was hidden 1 cm under the surface in the target quadrant. The animals were trained to find the hidden platform in four trials per day. Rats and mice that failed to find the hidden platform within 60 and 120 s were directed to stay on the platform for 20 s before being returned to their holding cage. Spatial memory was measured 24 h after the last training trial, and the hidden platform was removed. The Any-maze Tracking System (Stoelting, USA) was used to record and analyze latencies and swimming distances.

### Statistical analysis

All data are presented as the mean ± SEM. Escape latency during MWM training and the time spent on the rotarod during the rotarod test were analyzed by Two-way ANOVA. All the other data were analyzed by one-way ANOVA. Statistical significance was set at p < 0.05.

## Results

### HI activates the NLRP3 inflammasome signaling pathway

**Fig. 1:**
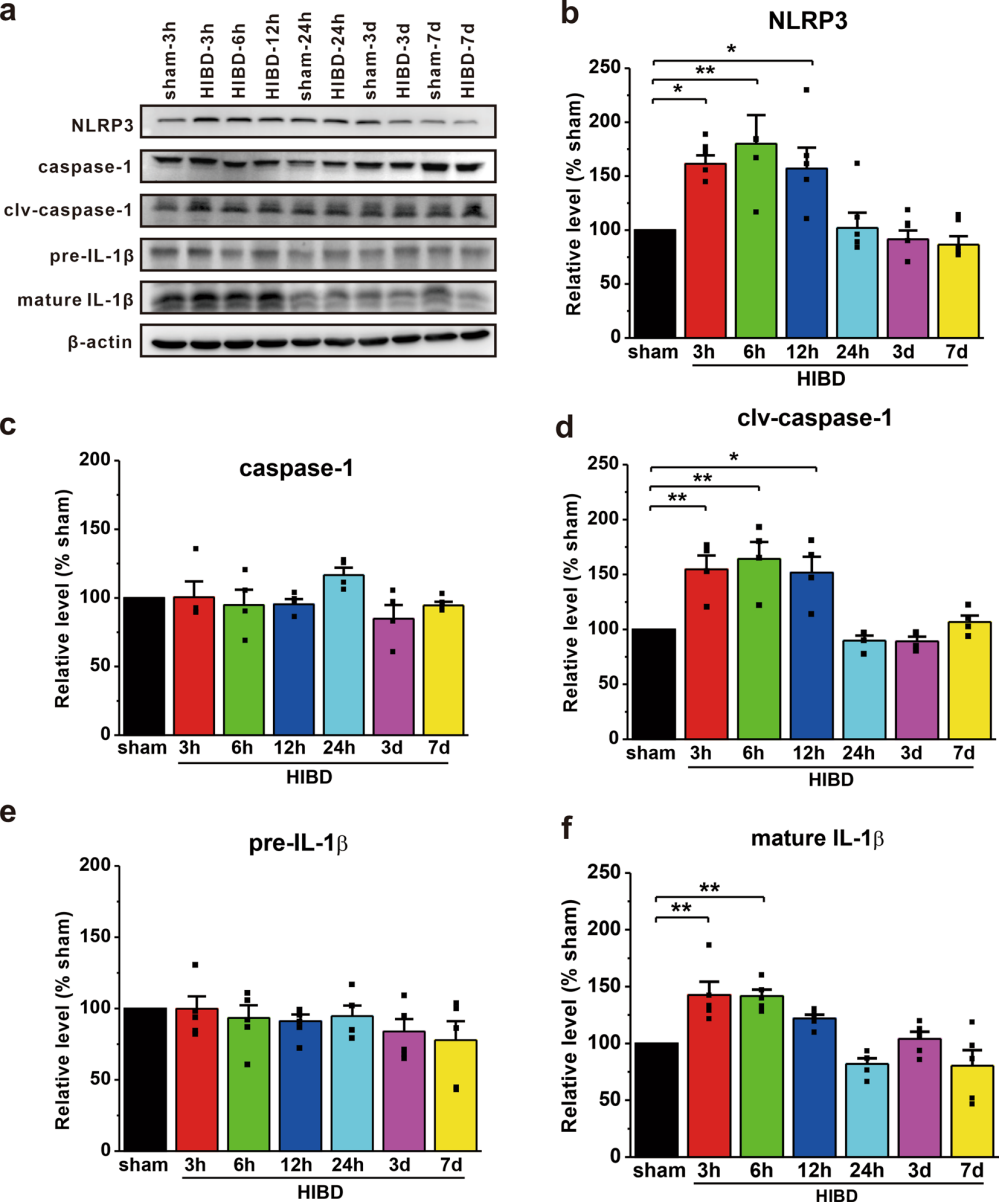
The NLRP3 inflammasome signaling pathway is activated in rats with HIBD. **a** The expression of NLRP3 inflammasome proteins in the brain tissues of rats with HIBD was detected by Western blotting. **b** The expression of NLRP3 showed an inverted U-shape and peaks at 6 h following HIBD. **c**–**f** The protein levels of clv-caspase-1 (**d**) and mature IL-1β (**f**), but not caspase-1 (**c**) and pre-IL-1β (**e**), were significantly increased in rats with HIBD compared with sham rats. The data were presented as the mean ± SEM. *p < 0.05, **p < 0.01

Brain tissues were collected at different time points following HIBD to detect the activation of the NLRP3 inflammasome pathway. As shown in Fig. 1, a significant increase in the expression of NLRP3 inflammasome proteins, including NLRP3 (HIBD-3 h: 161.4 ± 7.9%, p = 0.021 vs. sham; HIBD-6 h: 180.1 ± 26.5%, p = 0.004 vs. sham; HIBD-12 h: 157.1 ± 19.4%, p = 0.030 vs. sham; n = 5; Fig. 1a,  b), cleaved caspase-1 (clv-caspase-1) (HIBD-3 h: 154.6 ± 12.6%, p = 0.009 vs. sham; HIBD-6 h: 164.2 ± 15.4%, p = 0.003 vs. sham; HIBD-12 h: 151.6 ± 14.6%, p = 0.012 vs. sham; n = 4; Fig. 1a, d), and mature IL-1β (HIBD-3 h: 142.7 ± 11.5%, p = 0.002 vs. sham; HIBD-6 h: 141.7 ± 5.6%, p = 0.002 vs. sham; n = 5; Fig. 1a, f), was observed in rats with HIBD during the early stage of ischemia. No significant differences in caspase-1 (n = 4; Fig. 1a, c) or pre-IL-1β (n = 5; Fig. 1a, e) were observed among these groups.

**Fig. 2:**
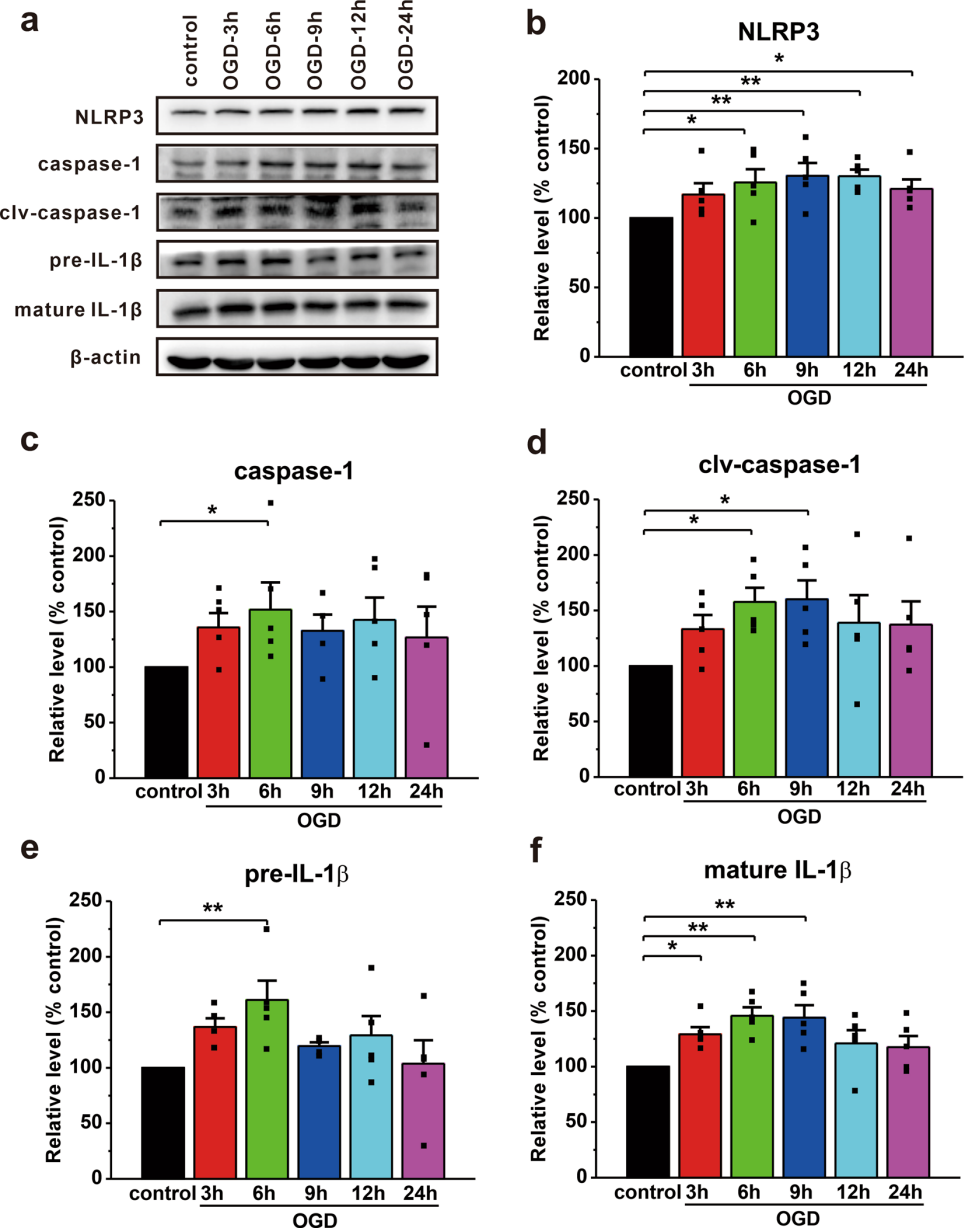
The NLRP3 inflammasome signaling pathway is activated in cultured primary neurons subjected to OGD.** a** The expression of NLRP3 inflammasome proteins in cultured neurons after OGD was detected by Western blotting. **b** The expression of NLRP3 showed an inverted U-shape and peaks at 9 h following OGD. **c**–**f** The protein levels of caspase-1 (**c**), clv-caspase-1 (**d**), pre-IL-1β (**e**), and mature IL-1β (**f**) were significantly increased in neurons following OGD in comparison with the control. The data were presented as the mean ± SEM. *p < 0.05, **p < 0.01

We then assessed NLRP3 inflammasome proteins in cultured primary neurons subjected to OGD to determine if ischemia-like conditions activated the NLRP3 inflammasome pathway in vitro. Similar to the in vivo findings, OGD significantly increased the expression of inflammasome proteins including NLRP3 (OGD-6 h: 125.6 ± 9.6%, p = 0.020 vs. control; OGD-9 h: 130.4 ± 9.3%, p = 0.007 vs. control; OGD-12 h: 130.2 ± 4.7%, p = 0.007 vs. control; OGD-24 h: 120.9 ± 6.8%, p = 0.050 vs. control; n = 5; Fig. 2a, b), caspase-1 (OGD-6 h: 151.7 ± 24.6%, p = 0.050 vs. control; n = 5; Fig. 2a, c), clv-caspase-1 (OGD-6 h: 157.6 ± 12.9%, p = 0.023 vs. control; OGD-9 h: 160.1 ± 16.9%, p = 0.018 vs. control; n = 5; Fig. 2a, d), pre-IL-1β (OGD-6 h: 161.1 ± 17.4%, p = 0.004 vs. control; n = 5; Fig. 2a, e) and mature IL-1β (OGD-3 h: 129.1 ± 6.5%, p = 0.015 vs. control; OGD-6 h: 145.7 ± 7.7%, p = 0.001 vs. control; OGD-9 h: 144 ± 11.2%, p = 0.001 vs. control; n = 5; Fig. 2a, f) in cultured neurons.

### Pharmacological inhibition of caspase-1 increases surface expression of GluA1

**Fig. 3:**
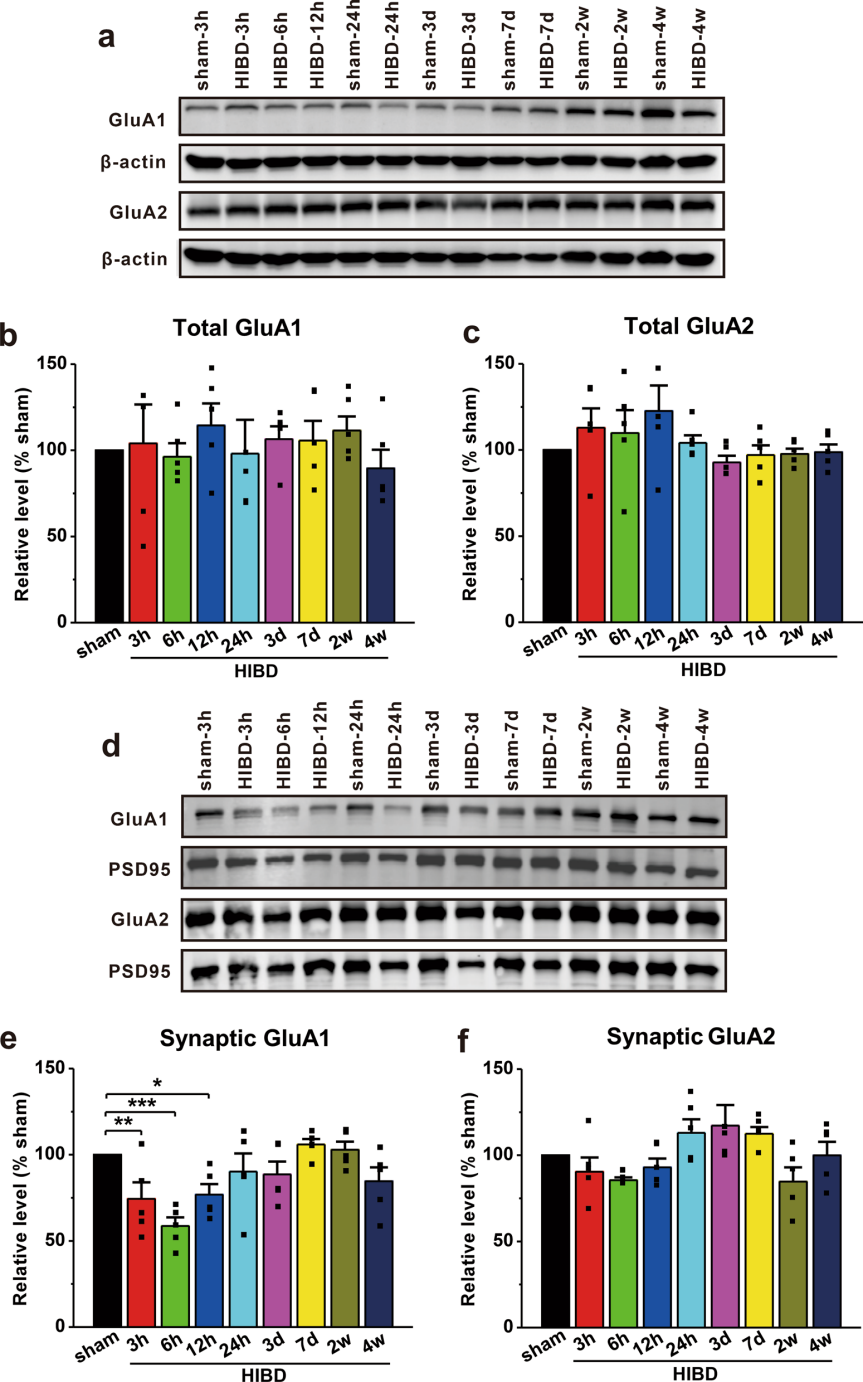
HIBD decreases the expression of synaptic GluA1.** a** The expression of cytoplasmic AMPARs in the brain tissues of rats with HIBD was detected by a Western blotting. **b**, **c** The total protein levels of GluA1 (**b**) and GluA2 (**c**) in rats with HIBD remained unchanged in comparison with those in sham rats. **d** Western blotting was used to detect the expression of synaptic AMPARs in the brain tissues of rats with HIBD. **e**, **f** The expression of GluA1 (**e**), but not GluA2 (**f**), was notably decreased in the synaptosomal fraction in rats with HIBD compared with sham rats. The data were presented as the mean ± SEM. *p < 0.05, **p < 0.01, ***p < 0.001

Glutaminergic excitotoxicity is primarily mediated by extracellular glutamate receptors, including NMDARs and AMPARs, and is thought to play an important role in HI-induced cell death [3, 24, 25]. Therefore, we next examined the alterations in AMPAR subunits including GluA1 and GluA2 in postsynaptic densities and total tissue lysates under ischemia-like conditions. The results showed that neither GluA1 nor GluA2 was changed in total tissue lysates (Fig. 3a–c). However, a specific decrease in the synaptic amount of GluA1 (HIBD-3 h: 74.4 ± 9.6%, p = 0.010 vs. sham; HIBD-6 h: 58.7 ± 5.0%, p < 0.001 vs. sham; HIBD-12 h: 76.8 ± 6.1%, p = 0.018 vs. sham; n = 5; Fig. 3d, e), but not GluA2 (Fig. 3d, f), was observed following HIBD. These data suggest that alterations in the AMPAR GluA1 subunit may have a significant impact on HI-induced cell death.

**Fig. 4:**
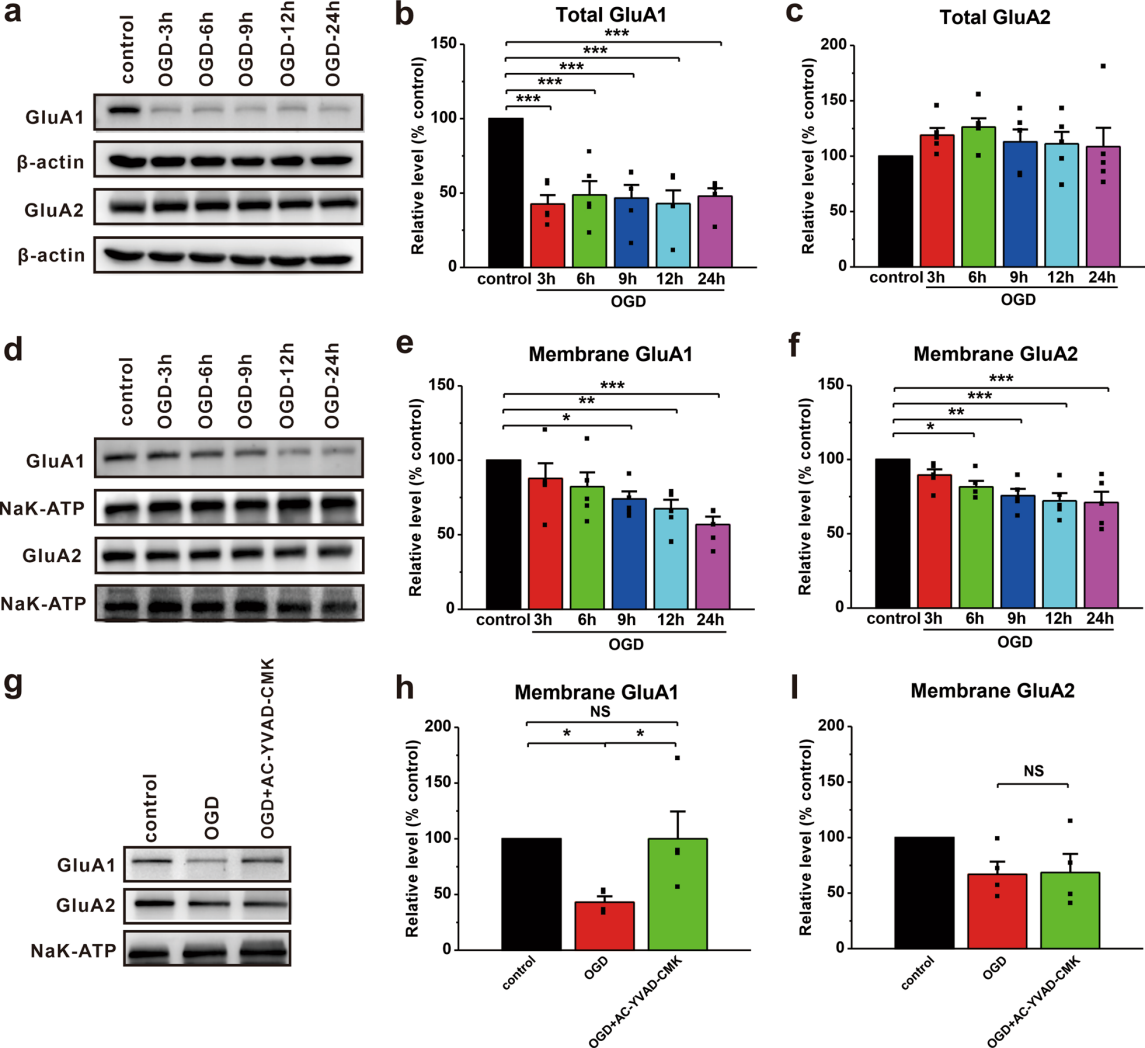
Inhibiting caspase-1 prevents the OGD-induced decrease in GluA1 expression in the plasma membrane.** a** Western blotting was used to detect the expression of cytoplasmic AMPARs in cultured neurons following OGD. **b**, **c** The expression of GluA1 (**b**), but not GluA2 (**c**), was notably decreased in the total cell lysates of cultured neurons following OGD. **d** Western blotting was used to detect the expression of AMPARs in the plasma membrane of cultured neurons following OGD. **e**, **f** The expression of GluA1 (**e**) and GluA2 (**f**) were notably decreased in the plasma membrane of cultured neurons following OGD. **g** The expression of AMPARs in the plasma membrane of cultured neurons treated with AC-YVAD-CMK (5 µM) 1 h before OGD was detected by Western blotting. **h**, **i** The expression of GluA1 (**h**), but not GluA2 (**i**), was restored in the plasma membrane of cultured neurons after AC-YVAD-CMK treatment. The data were presented as the mean ± SEM. *p < 0.05, **p < 0.01, ***p < 0.001

Next, AC-YVAD-CMK, which is a specific inhibitor of caspase-1, was used to investigate the correlation between the NLRP3 inflammasome pathway and GluA1 localization. The results showed that OGD treatment significantly decreased the expression of GluA1 in total cell lysates (OGD-3 h: 42.7 ± 6.0%, p < 0.001 vs. control; OGD-6 h: 48.7 ± 9.3%, p < 0.001 vs. control; OGD-9 h: 46.6 ± 8.9%, p < 0.001 vs. control; OGD-12 h: 42.9 ± 9.0%, p < 0.001 vs. control; OGD-24 h: 47.9 ± 5.4%, p < 0.001 vs. control; n = 5; Fig. 4a and b) and the plasma membrane (OGD-9 h: 74.0 ± 5.1%, p = 0.013 vs. control; OGD-12 h: 67.4 ± 6.0%, p = 0.003 vs. control; OGD-24 h: 56.8 ± 5.3%, p < 0.001 vs. control; n = 6; Fig. 4d, e). The expression of GluA2 in the plasma membrane (OGD-6 h: 81.6 ± 4.0%, p = 0.011 vs. control; OGD-9 h: 75.6 ± 4.6%, p = 0.001 vs. control; OGD-12 h: 72.2 ± 5.2%, p < 0.001 vs. control; OGD-24 h: 71.0 ± 7.3%, p < 0.001 vs. control; n = 6; Fig. 4d, f), but not in total cell lysates (Fig. 4a, c), was decreased in the neurons following OGD treatment. Importantly, we found that AC-YVAD-CMK almost completely prevented the OGD-induced reduction in GluA1 (OGD: 43.1 ± 5.2%, p = 0.021 vs. control; OGD + AC-YVAD-CMK: 100 ± 24.5%, p = 0.022 vs. OGD; n = 4; Fig. 4g, h) in the plasma membrane, but did not affect GluA2 (OGD: 66.9 ± 11.4%, p = 0.077 vs. control; OGD + AC-YVAD-CMK: 68.6 ± 16.8%, p = 0.090 vs. control; n = 4; Fig. 4g, i).

### Pharmacological inhibition of caspase-1 reduces the interaction between p97 and GluA1

**Fig. 5:**
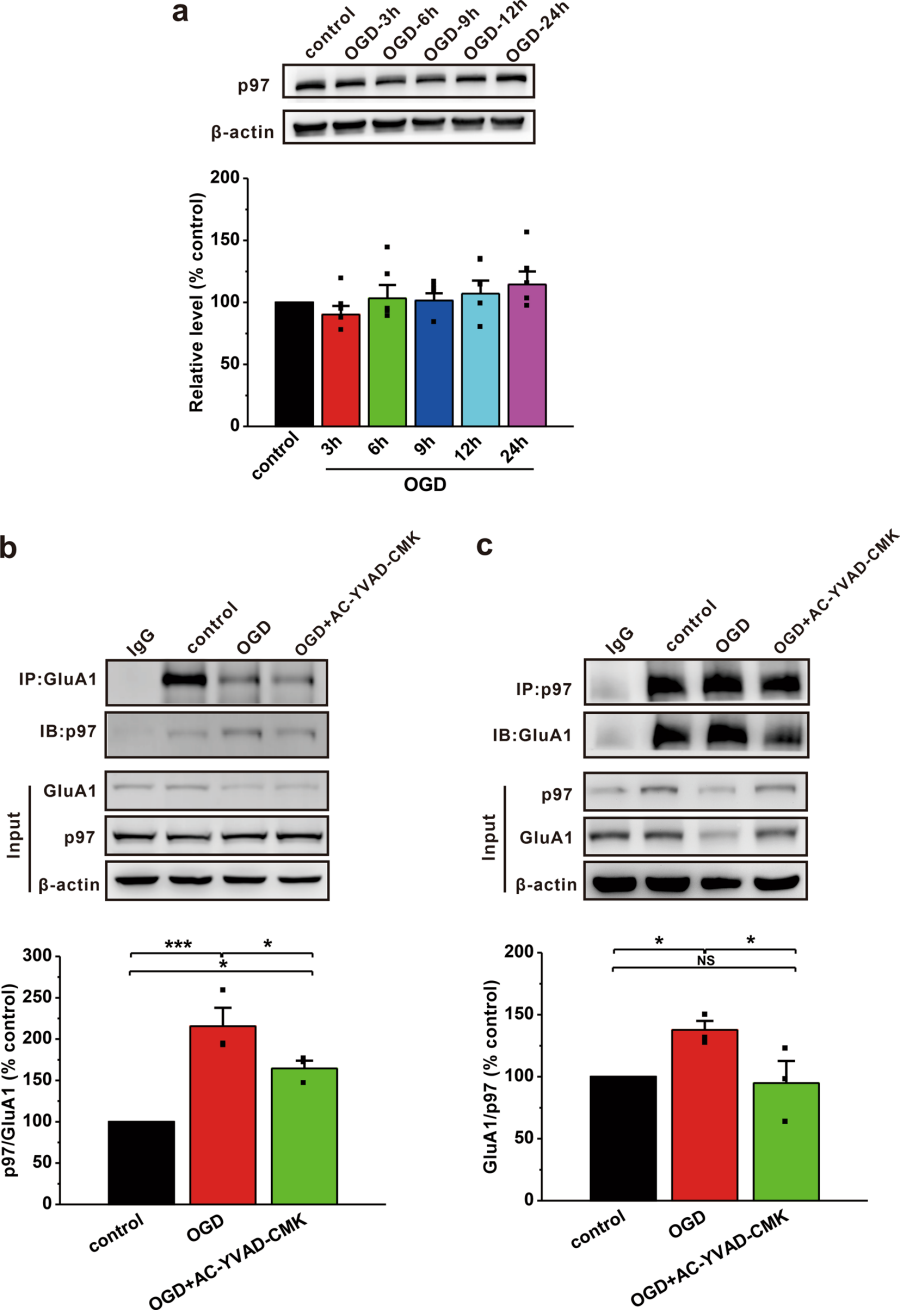
Inhibiting caspase-1 promotes the dissociation of p97 from GluA1 in cultured primary neurons subjected to OGD.** a** The expression of p97 in cultured primary neurons remained unchanged following OGD. **b**, **c** Co-IP assays were performed 6 h after OGD in cultured neurons. The interaction between p97 and GluA1 was enhanced in neurons subjected to OGD compared to control neurons. AC-YVAD-CMK led to the dissociation of p97 from GluA1. The data were presented as the mean ± SEM. *p < 0.05, ***p < 0.001

Our recent study has reported that the ATPase valosin-containing protein (VCP/p97) specifically interacts with GluA1 and regulates GluA1-homo AMPAR formation and trafficking [45]. We next wanted to determine the role of p97 in the NLRP3 inflammasome-induced reduction in synaptic GluA1 under OGD conditions. The results showed that OGD had no effect on p97 expression in total cell lysates (OGD-3 h: 90.2 ± 6.9%, p = 0.413 vs. control; OGD-6 h: 103.3 ± 10.7%, p = 0.781 vs. control; OGD-9 h: 101.5 ± 5.8%, p = 0.902 vs. control; OGD-12 h: 107.1 ± 10.5%, p = 0.552 vs. control; OGD-24 h: 114.4 ± 10.5%, p = 0.233 vs. control; n = 5; Fig. 5a). Consistent with our recent report [45], we found that p97 co-immunoprecipitated with GluA1 (n = 3; Fig. 5b), but not GluA2 (Additional file 1: Fig. S2). To further confirm the finding that p97 interacted with GluA1, we used a GluA1 antibody to precipitate p97 and obtained a similar result that GluA1 was able to co-immunoprecipitate with p97 (n = 3; Fig. 5c). More importantly, the interaction between p97 and GluA1 was increased in cultured neurons subjected to OGD, and AC-YVAD-CMK treatment dramatically reduced this interaction (Fig. 5b, c). Collectively, these data suggest that OGD-induced NLRP3 inflammasome activation promotes the formation of the p97 and GluA1 complex. Inhibiting caspase-1 with AC-YVAD-CMK may increase GluA1 trafficking from the intracellular reserve pool to the membrane by dissociating p97 from the GluA1 complex.

### Pharmacological inhibition of caspase-1 alleviates behavioral deficits in rats with HIBD

**Fig. 6:**
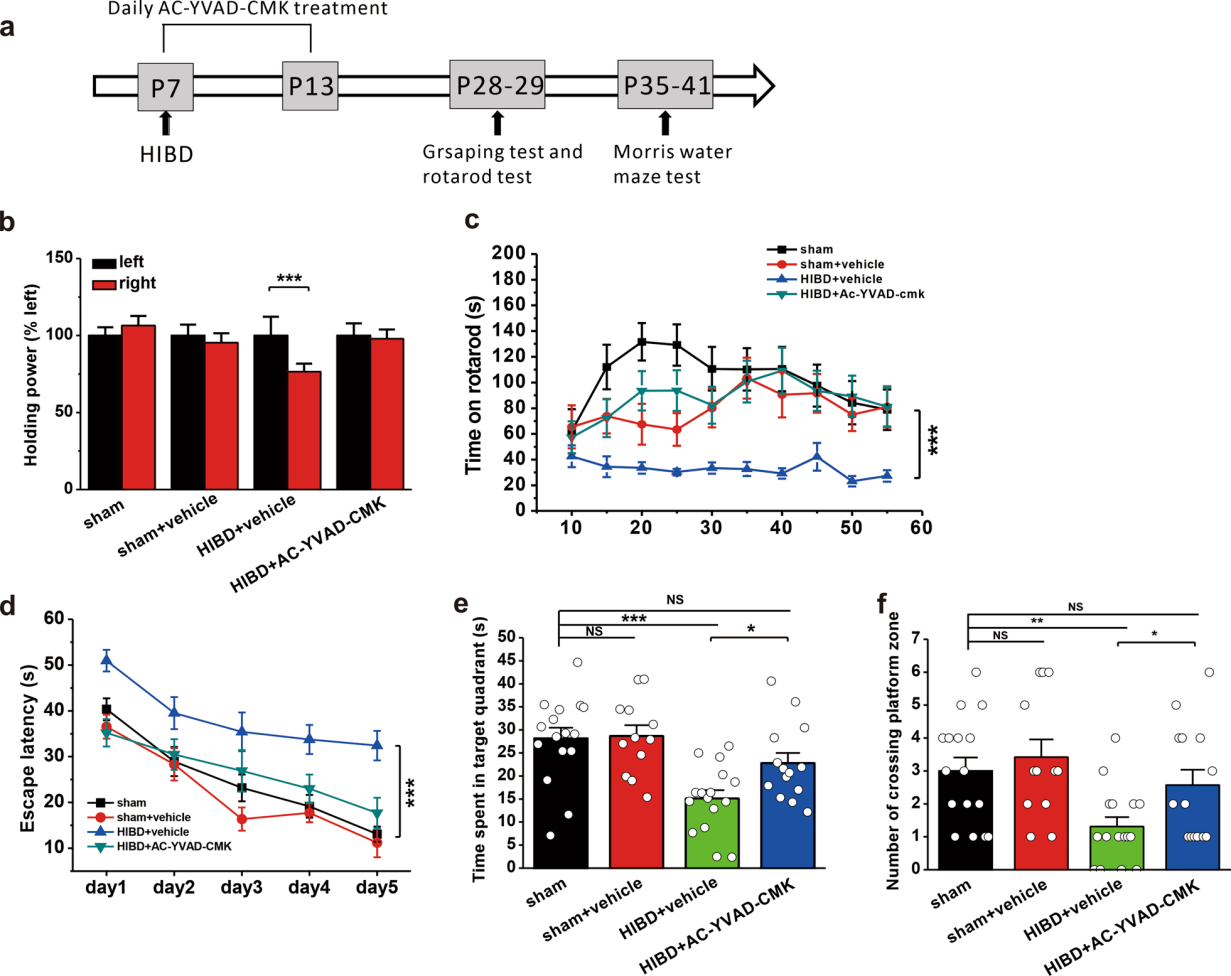
Pharmacological inhibition of caspase-1 by AC-YVAD-CMK prevents HIBD-induced behavioral deficits in rats.** a** The experimental protocols of HIBD establishment and AC-YVAD-CMK (1 mg/kg, i.p.) injections as well as behavioral tests. **b** AC-YVAD-CMK treatment increased holding power of right forelimb during the grasping test in rats with HIBD. **c** AC-YVAD-CMK treatment of rats with HIBD increased the time spent on the rod during the rotarod test. **d**–**f** AC-YVAD-CMK treatment alleviated HIBD-induced spatial learning and memory deficits in the MWM test. AC-YVAD-CMK treatment of rats with HIBD shortened the latency to find the hidden platform (**d**) during training, and increased the time spent in the target quadrant (**e**) and the number of entries into the platform zone (**f**) during memory retrieval. The data were presented as the mean ± SEM. *p < 0.05, **p < 0.01, ***p < 0.001

A previous study has demonstrated that GluA1-homo AMPARs play critical roles in mediating long-term potentiation (LTP) and cognitive function [46]. Our findings suggested that inhibiting caspase-1 could prevent the OGD-induced reduction in GluA1 in the plasma membrane (Fig. 4). Therefore, we conducted three different behavioral tests to evaluate the influence of AC-YVAD-CMK on HI-induced deficits in motor, learning and memory functions in rats with HIBD (Fig. 6a).

In the grasping test, right forelimb grip strength was significantly reduced compared to that of the left forelimb in rats with HIBD (HIBD + vehicle: n = 15, p < 0.001; Fig. 6b), but not in sham rats (sham: n = 16, p = 0.734; sham + vehicle: n = 12, p = 0.952; Fig. 6b). As expected, AC-YVAD-CMK treatment of rats with HIBD resulted in significant recovery of right forelimb grip strength (HIBD + AC-YVAD-CMK: n = 16, p = 0.348; Fig. 6b). In the rotarod test, the latency to fall from the rod was significantly shorter in rats with HIBD than in sham rats (sham: n = 15; sham + vehicle: n = 12; HIBD + vehicle: n = 15, p < 0.001 vs. sham, p = 0.002 vs. sham + vehicle; Fig. 6c). AC-YVAD-CMK treatment restored the time on the rotarod to the sham level (HIBD + AC-YVAD-CMK: n = 15, p = 0.249 vs. sham, p = 0.566 vs. sham + vehicle, p < 0.001 vs. HIBD + vehicle; Fig. 6c).

Previous findings in animal models and humans revealed that the major complication of HI was learning and memory deficits [47, 48]. Therefore, we used the MWM model to explore whether AC-YVAD-CMK could alleviate HI-induced spatial learning and memory impairments in rats with HIBD. The results showed that rats with HIBD spent more time finding the hidden platform during the spatial training period than sham rats (sham: n = 16; sham + vehicle: n = 12; HIBD + vehicle: n = 16, p < 0.001 vs. sham, p < 0.001 vs. sham + vehicle; Fig. 6d), indicating impaired spatial learning ability in response to ischemic injury. Compared to vehicle treatment, AC-YVAD-CMK treatment significantly decreased the latency to find the hidden platform in rats with HIBD, indicating a significant improvement in spatial learning (HIBD + AC-YVAD-CMK: n = 14, p = 0.579 vs. sham, p = 0.168 vs. sham + vehicle, p < 0.001 vs. HIBD + vehicle; Fig. 6d). Furthermore, AC-YVAD-CMK treatment dramatically increased the time spent in the target quadrant (sham: 28.1 ± 2.3 s; sham + vehicle: 28.7 ± 2.4s; HIBD + vehicle: 15.1 ± 1.8 s, p < 0.001 vs. sham, p < 0.001 vs. sham + vehicle; HIBD + AC-YVAD-CMK: 22.8 ± 2.2 s, p = 0.084 vs. sham, p = 0.088 vs. sham + vehicle, p = 0.015 vs. HIBD + vehicle; Fig. 6e) and the number of entries into the platform zone (sham: 3.0 ± 0.4; sham + vehicle: 3.4 ± 0.5; HIBD + vehicle: 1.3 ± 0.3, p = 0.004 vs. sham, p = 0.001 vs. sham + vehicle; HIBD + AC-YVAD-CMK: 2.6 ± 0.5, p = 0.467 vs. sham, p = 0.184 vs. sham + vehicle, p = 0.036 vs. HIBD + vehicle; Fig. 6f) during the spatial memory test in rats with HIBD, indicating a great improvement in spatial memory retrieval.

Taken together, the present data demonstrate that blocking the NLRP3 inflammasome pathway with the caspase-1 inhibitor AC-YVAD-CMK can prevent HI-induced motor and cognitive dysfunction.

### Genetic inhibition of the NLRP3 inflammasome pathway alleviates behavioral deficits in mice with HIBD

**Fig. 7:**
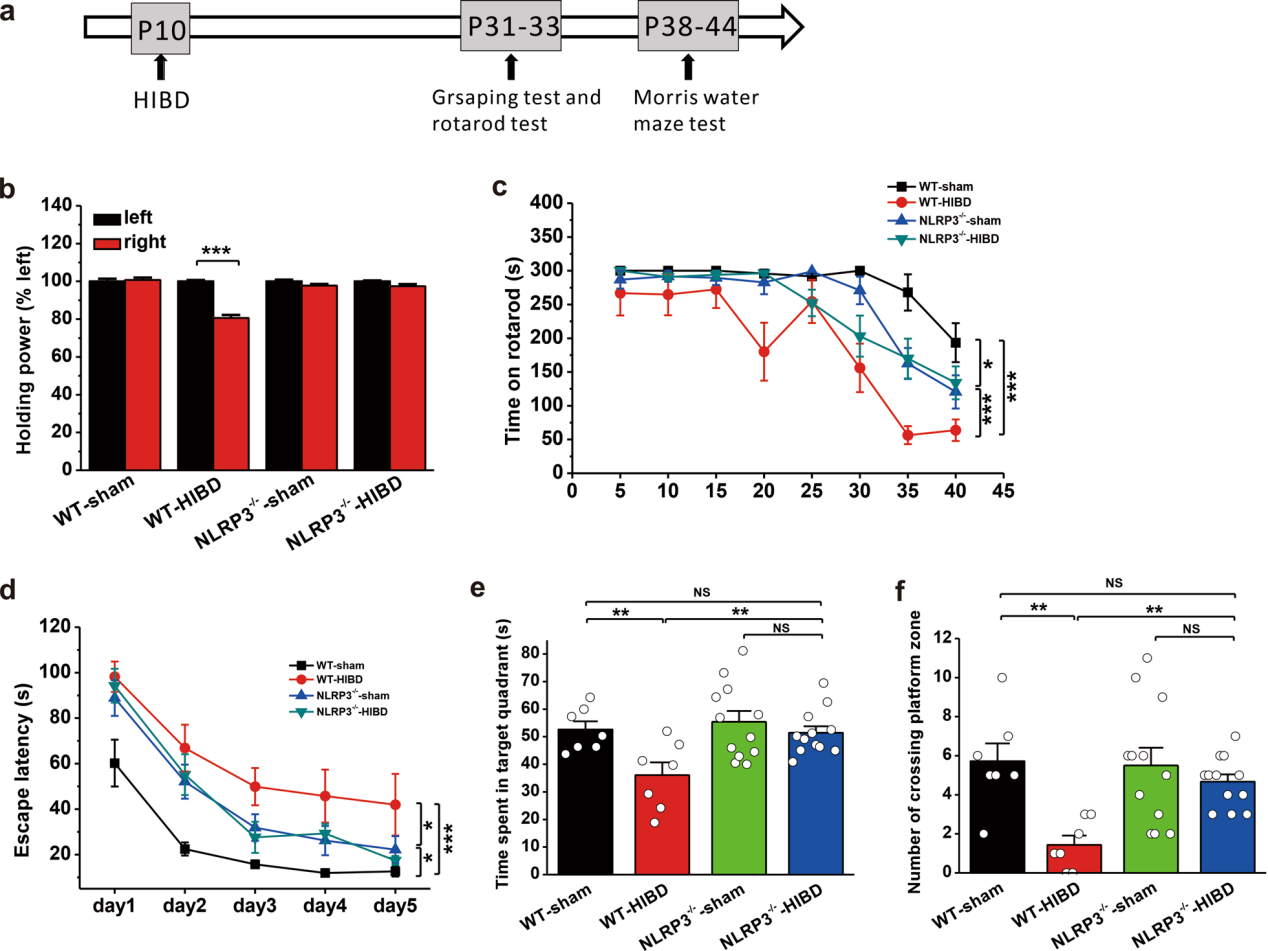
Genetic ablation of NLRP3 prevents HIBD-induced behavioral deficits in mice.** a** Experimental protocols for model establishment and behavioral tests. **b** Genetic ablation of NLRP3 (NLRP3^−/−^) improved HIBD-induced impairment of the holding power of the right forelimb during the grasping test. **c** The time spent on the rod was significantly longer in NLRP3^−/−^ mice following HIBD than in WT mice during the rotarod test. **d**–**f** Genetic ablation of NLRP3 (NLRP3^−/−^) improved HIBD-induced impairment of spatial learning and memory during the MWM test. HIBD shortened the latency of NLRP3^−/−^ mice to find the hidden platform during spatial learning (**d**), compared with WT mice. Genetic ablation of NLRP3 (NLRP3^−/−^) increased the time spent in the target quadrant (**e**) and the number of entries into the platform zone (**f**). The data were presented as the mean ± SEM. *p < 0.05, **p < 0.01, ***p < 0.001

**Fig. 8:**
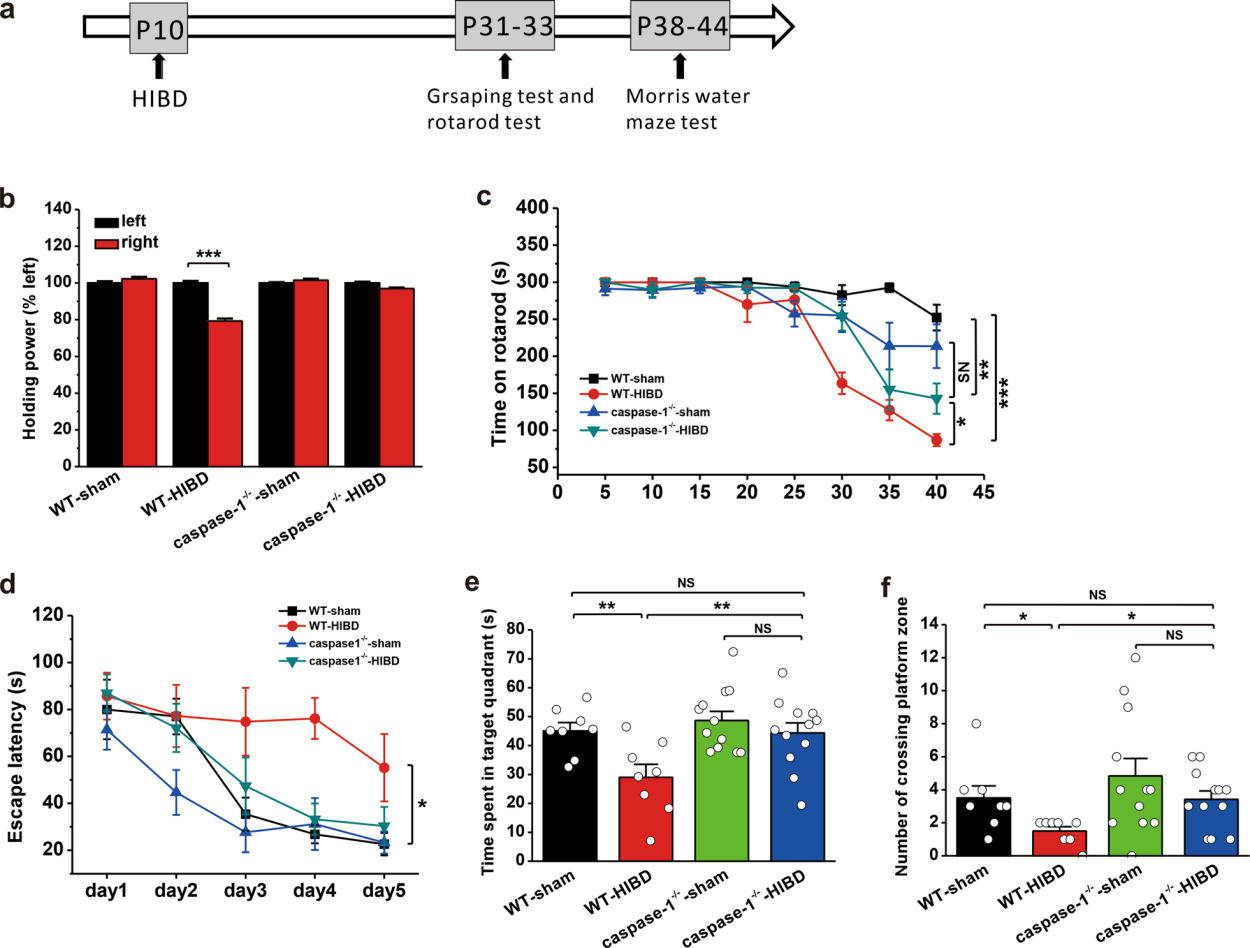
Genetic ablation of caspase-1 prevents HIBD-induced behavioral deficits in mice.** a** Experimental protocols for model establishment and behavioral tests. **b** Genetic ablation of caspase-1 (caspase-1^−/−^) improved HIBD-induced impairment of the holding power of the right forelimb during the grasping test. **c** The time spent on the rod was significantly longer in caspase-1^−/−^ mice following HIBD than in WT mice during the rotarod test. **d**–**f** Genetic ablation of caspase-1 (caspase-1^−/−^) improved HIBD-induced impairments in spatial learning and memory during the MWM test. In caspase-1^−/−^ mice, HIBD shortened the latency to find the hidden platform during spatial learning (**d**) compared with WT mice. Genetic ablation of caspase-1 (caspase-1^−/−^) increased the time spent in the target quadrant (**e**) and the number of entries into the platform zone (**f**). The data were presented as the mean ± SEM. *p < 0.05, **p < 0.01, ***p < 0.001

To further confirm the influence of the NLRP3 inflammasome pathway on behavioral deficits caused by HI, the motor and cognitive functions of NLRP3^−/−^ (Fig. 7a) and caspase-1^−/−^ (Fig. 8a) mice were examined. In the grasping test, right forelimb grip strength was significantly increased in NLRP3^−/−^ (WT-sham: n = 7, p = 0.758; WT-HIBD: n = 7, p < 0.001; NLRP3^−/−^-sham: n = 12, p = 0.228; NLRP3^−/−^-HIBD: n = 12, p = 0.174; Fig. 7b) and caspase-1^−/−^ (WT-sham: n = 8, p = 0.276; WT-HIBD: n = 8, p < 0.001; caspase-1^−/−^-sham: n = 12, p = 0.405; caspase-1^−/−^-HIBD: n = 14, p = 0.081; Fig. 8b) mice following HIBD compared to those in WT mice, indicating improvements in myodynamia. In the rotarod test, compared to WT mice, NLRP3^−/−^ (WT-sham: n = 7; WT-HIBD: n = 7, p < 0.001 vs. WT-sham; NLRP3^−/−^-sham: n = 12, p = 0.032 vs. WT-sham, p < 0.001 vs. WT-HIBD; NLRP3^−/−^-HIBD: n = 12, p = 0.010 vs. WT-sham, p < 0.001 vs. WT-HIBD, p = 0.504 vs. NLRP3^−/−^-sham; Fig. 7c) and caspase-1^−/−^ (WT-sham: n = 8; WT-HIBD: n = 8, p < 0.001 vs. WT-sham; caspase-1^−/−^-sham: n = 12, p = 0.017 vs. WT-sham, p = 0.002 vs. WT-HIBD; caspase-1^−/−^-HIBD: n = 14, p = 0.001 vs. WT-sham, p = 0.020 vs. WT-HIBD, p = 0.281 vs. caspase-1^−/−^-sham; Fig. 8c) mice spent much more time on the rotarod following HIBD, indicating a significant improvement in motor coordination.

Compared to WT mice, NLRP3^−/−^ (WT-sham: n = 7; WT-HIBD: n = 7, p < 0.001 vs. WT-sham; NLRP3^−/−^-sham: n = 12, p = 0.010 vs. WT-sham, p = 0.027 vs. WT-HIBD; NLRP3^−/−^-HIBD: n = 12, p = 0.010 vs. WT-sham, p = 0.933 vs. NLRP3^−/−^-sham, p = 0.032 vs. WT-HIBD; Fig. 7d) and caspase-1^−/−^ (WT-sham: n = 8; WT-HIBD: n = 8, p < 0.001 vs. WT-sham; caspase-1^−/−^-sham: n = 12, p = 0.017 vs. WT-sham, p = 0.002 vs. WT-HIBD; caspase-1^−/−^-HIBD: n = 12, p = 0.001 vs. WT-sham, p = 0.020 vs. WT-HIBD, p = 0.281 vs. caspase-1^−/−^-sham; Fig. 8d) mice exhibited significant improvements in spatial learning following HIBD because they spent much less time searching for the hidden platform in the MWM test. During the spatial memory test, the time spent in the target quadrant (WT-sham: 52.6 ± 3.0 s; WT-HIBD: 36.1 ± 4.6 s, p = 0.008 vs. WT-sham; NLRP3^−/−^-sham: 55.4 ± 4.0 s, p = 0.596 vs. WT-sham, p = 0.001 vs. WT-HIBD; NLRP3^−/−^-HIBD: 51.4 ± 2.4 s, p = 0.820 vs. WT-sham, p = 0.006 vs. WT-HIBD, p = 0.380 vs. NLRP3^−/−^-sham; Fig. 7e) and the number of entries into the platform zone (WT-sham: 5.7 ± 0.9; WT-HIBD: 1.4 ± 0.5, p = 0.001 vs. WT-sham; NLRP3^−/−^-sham: 5.5 ± 0.9, p = 0.843 vs. WT-sham, p = 0.001 vs. WT-HIBD; NLRP3^−/−^-HIBD: 4.7 ± 0.4, p = 0.335 vs. WT-sham, p = 0.005 vs. WT-HIBD, p = 0.372 vs. NLRP3^−/−^-sham; Fig. 7f) were significantly increased in NLRP3^−/−^ mice following HIBD compared to WT mice. Similarly, caspase-1^−/−^ mice with HIBD showed improved spatial memory compared to WT mice, which was reflected by dramatic increases in the time spent in the target quadrant (WT-sham: 45.1 ± 2.9 s; WT-HIBD: 29.0 ± 4.5 s, p = 0.007 vs. WT-sham; caspase-1^−/−^-sham: 48.7 ± 3.2 s, p = 0.487 vs. WT-sham, p < 0.001 vs. WT-HIBD; caspase-1^−/−^-HIBD: 44.3 ± 3.5 s, p = 0.884 vs. WT-sham, p = 0.005 vs. WT-HIBD, p = 0.349 vs. caspase-1^−/−^-sham; Fig. 8e) and the number of entries into the platform zone (WT-sham: 3.5 ± 0.7; WT-HIBD: 1.5 ± 0.3, p = 0.050 vs. WT-sham; caspase-1^−/−^-sham: 4.8 ± 1.1, p = 0.244 vs. WT-sham, p = 0.005 vs. WT-HIBD; caspase-1^−/−^-HIBD: 3.4 ± 0.5, p = 0.941 vs. WT-sham, p = 0.050 vs. WT-HIBD, p = 0.168 vs. caspase-1^−/−^-sham; Fig. 8f).

Taken together, these findings demonstrate that blocking the NLRP3 inflammasome pathway via genetic ablation of NLRP3 or caspase-1 can alleviate HI-induced impairments in myodynamia, motor coordination, and spatial learning and memory.

## Discussion

Brain injury caused by HI can persist for hours to weeks [49]. Mitochondrial energy failure, excitotoxicity, inflammation, calcium overload and oxidative stress are the most extensively studied pathogenic mechanisms in HIBD [3, 28]. The early stage of cerebral ischemia is characterized by a decrease in cerebral blood flow, thereby reducing intracellular ATP levels and leading to cell membrane depolarization and excessive glutamate release. The overactivation of glutamate receptors eventually results in cell edema and dissolution [28, 50]. Few treatments can effectively minimize brain injury caused by the initial energy failure that lasts from a few minutes to hours [6, 51, 52]. However, there is a potential damage period that usually occurs 1–6 h after HI that is between the first and second energy failures [52, 53], indicating that targeted interventions could minimize excitotoxicity, inflammation, and oxidative stress-related cell damage [3, 53].

A growing body of evidence has revealed that innate immunity is triggered a few minutes after HI, leading to the release of inflammatory cytokines including IL-1 and TNF-α by neurons, astrocytes, and microglia [54–57]. Antagonists of the IL-1β receptor can alleviate inflammation-related brain injury caused by HI [58]. Furthermore, previous studies have revealed time-dependent activation of the NLRP3 inflammasome pathway in ischemic stroke [18, 19]. In line with these reports, our results demonstrate that the NLRP3 inflammasome pathway, including caspase-1 and IL-1β, is time-dependently activated under ischemia-like conditions in vivo and in vitro (Figs. 1 and 2). Considering the pathological progression of HIBD, secondary energy failure occurs approximately 6 h after HI [3, 52, 53]. Based on the inverted U-shape increase in the NLRP3 inflammasome proteins (Figs. 1 and 2), we hypothesize that inflammation may play a critical role primarily in the early phase of HIBD rather than in secondary energy failure stage. Since caspase-1 acts as a crucial mediator of the NLRP3 inflammasome pathway, we used a caspase-1 inhibitor to block the NLRP3 inflammasome pathway. Although there are various caspase-1 inhibitors, AC-YVAD-CMK was selected because of its superior specificity compared with other inhibitors such as VX-765 [59–61]. Here, we reported that pharmacological intervention immediately after HIBD or genetic ablation of caspase-1 improved HI-induced myodynamia, motor coordination, learning, and memory impairment in animal models (Figs. 6 and 8). In addition, the neuroprotective effect of AC-YVAD-CMK was also evident at the histopathological level (Additional file 1: Fig. S3), which was consistent with the functional outcomes. Notably, although inhibiting the NLRP3 inflammasome pathway with AC-YVAD-CMK promoted neurological recovery following HIBD, its potential side effects need to be further explored in future studies.

If interventions fail during the potential damage period, excitotoxicity increases and becomes the dominant factor during secondary energy failure [62–64]. Clinical evidence has shown that secondary energy failure can result in moderate to severe neonatal HIE, causing a number of major neurological sequelae, such as cerebral palsy [4, 5, 65]. Excitotoxicity appears to play a critical role in the pathological process of HIBD [3, 53], and AMPAR regulation has the potential to minimize ischemic brain injury [37]. Additionally, the immature brain exhibits higher sensitivity to excitotoxic insults than the mature brain [66, 67], highlighting the necessity of exploring the potential mechanisms of AMPARs in HIBD. Previous studies have primarily focused on the roles of GluA2-containing AMPARs in HIBD [35, 68–70]. However, in the early stage of brain development, many synapses contain GluA2‑lacking AMPARs, and the expression of GluA2 subunits gradually increases in rats until 14 days after birth [69, 71, 72]. In contrast to GluA2, the GluA1 subunit is expressed predominantly at higher levels, especially during early brain development [71, 73, 74], indicating that GluA1 may be more important in HI-induced immature brain damage. Indeed, we found an obvious decrease in the synaptic levels of the GluA1 subunit, but not GluA2 in vivo (Fig. 3). In addition, cultured neurons exhibited low total and membrane GluA1 expression, which was similar to recent reports [75–78], whereas GluA2 was only decreased in the membrane (Fig. 4). Notably, several previous reports have shown that OGD increases GluA1 expression on the plasma membrane in cultured neurons and brain slices [79, 80]. In the current study, we selected 7-day cultured neurons to mimic immature brain injury during OGD, while previous studies used neurons cultured 14–17 days before OGD [79–81]. Therefore, the developmental stage of the subjects and other variations in the OGD conditions may at least partially account for the reported inconsistencies. The reduction in GluA2 in the membrane may be caused by phosphorylation-mediated endocytosis [35, 68, 82], but the underlying mechanism by which GluA1 levels are reduced still requires further exploration.

It has been widely reported that neuroinflammation impairs brain function, especially learning and memory, by altering membrane expression of AMPARs [37, 83–85]. However, whether activation of the NLRP3 inflammasome pathway contributes to the reduction in membrane GluA1 is not unclear. In this study, we used AC-YVAD-CMK to inhibit the NLRP3 inflammasome pathway in cultured primary neurons. As expected, AC-YVAD-CMK rescued the HI-induced reduction in GluA1 but not GluA2 in the plasma membrane (Fig. 4). VCP/p97, which is an ATPase, is widely present in the cytoplasm. Together with a network of cofactors, p97 plays a critical role in various cellular processes that maintain genomic stability and proteostasis [86, 87]. Importantly, abnormal expression of p97 is associated with many diseases including neurodegenerative diseases, amyotrophic lateral sclerosis and cancers [86, 88]. Since p97 has been reported to be involved in synaptic plasticity by regulating the formation and trafficking of GluA1-homo AMPARs [45], which is important for learning and memory [43, 46], p97 may play an essential role in HI-induced brain dysfunction by regulating the surface expression of the GluA1 subunit. Indeed, our findings demonstrate that the interaction between p97 and GluA1 is up-regulated after OGD (Fig. 5). AC-YVAD-CMK markedly promotes the dissociation of p97 from GluA1, resulting in the release of GluA1 subunits from the intracellular reserve pool and transport to the postsynaptic membrane under ischemia-like conditions (Fig. 5). Notably, we found that caspase-1 was involved in p97-mediated GluA1 subunit trafficking following HIBD, but the underlying mechanism remains unclear. Thus, future studies should focus on elucidating how NLRP3 inflammasome signaling pathway factors, such as caspase-1, affect p97-mediated GluA1 trafficking after HIBD, which may benefit the development of novel HIE therapies.

## Conclusions

In summary, our present data suggest that NLRP3 inflammasome pathway activation enhances the interaction between p97 and GluA1, thereby inducing a decrease in surface GluA1. Blocking the NLRP3 inflammasome pathway with the caspase-1 specific inhibitor AC-YVAD-CMK prevents motor and cognitive impairments in HIBD models. These findings establish a scientific foundation for the development of NLRP3 inflammasome pathway inhibitors, such as AC-YVAD-CMK, as potential therapeutic interventions to improve outcomes in HIBD patients. Further studies should explore the underlying mechanisms by which caspase-1 is involved in the formation of the p97-GluA1 complex. In addition, further investigation of the potential side effects and limitations of NLRP3 inflammasome pathway blockers is warranted to provide a basis for validating our findings in a clinical setting and exploring alternative approaches for HIE treatment.

### Supplementary Information

**Additional file 1: Figure S1.** The NLRP3^−/−^ and caspase-1^−/−^ mice were genotyped by PCR with reverse transcriptase using mouse tail-tip DNA and mixed primers. The protein levels of NLRP3 and caspase-1 were assessed by Western blot in the brain tissues from WT, NLRP3^−/−^ and caspase-1^−/−^ mice. **a** The results of PCR showed a band of 666 bp in WT mice, and a band of 850 bp in NLRP3^−/−^ mice. The results of Western blot showed no NLRP3 expression in NLRP3^−/−^ mice. **b** The results of PCR showed a band of 500 bp in WT mice, and a band of 300 bp in caspase-1^−/−^ mice. The results of Western blot showed no caspase-1 and clv-caspase-1 expression in caspase-1^−/−^ mice. **Figure S2.** Co-IP assays show no interaction between GluA2 and p97 in the primarily cultured neurons with or without OGD treatment. **Figure S3.** TTC staining shows an improvement of infarction caused by HI with the treatment of AC-YVAD-CMK. **a** Representative TTC staining coronal brain sections (2 mm). Sections are labeled as five different levels (level 1–level 5) along the anterior (A) to posterior (P) axis. **b** Quantification of the cerebral infarct area in brain sections (infarcted area ratio (%) = white infarcted area/brain slice area).

## Data Availability

All data generated or analyzed during this study were included either in this article methods section. Other data supporting the findings of this study are available from the corresponding author upon reasonable request.

## Abbreviations

HIE: Hypoxic-ischemic encephalopathy
HIBD: Hypoxic-ischemic brain damage
HI: Hypoxic-ischemia
NOD: Nucleotide-binding oligomeric domain
NLRP3: NOD-like receptor protein 3
Caspase-1: Cysteinyl aspartate specific proteinase-1
IL-1β: Interleukin-1β
IL-18: Interleukin-18
ASC: Apoptosis-related specific protein
P97 (VCP): Valosin-containing protein
AMPAR: α-Amino-3-hydroxy-5-methyl-4-isoxazole-propionicacid receptor
GluA1: Glutamate receptor 1
GluA2: Glutamate receptor 2
NMDAR: *N*-Methyl-d-aspartate receptor
KAR: Kainate receptor
OGD: Oxygen-glucose deprivation
PAMP: Pathogen-associated molecular pattern
PRR: Pattern recognition receptor
SD: Sprague-Dawley
WT: Wild type
PCR: Polymerase chain reaction
TTC: Triphenyltetrazolium chloride
PBS: Phosphate buffered saline
HBSS: Hank’s Balanced Salt Solution
EDTA: Ethylene Diamine Tetraacetic Acid
DMEM: Dulbecco’s Modified Eagle Medium
FBS: Fetal bovine serum
EBSS: Earle’s Balanced Salt Solution
SDS: Sodium dodecyl sulfate
PAGE: Polyacrylamide gel electrophoresis
PVDF: Polyvinylidene difluoride
IP: Immunoprecipitation
MWM: Morris water maze
