# Supplementary material for: Inhibiting NLRP3 inflammasome signaling pathway promotes neurological recovery following hypoxic-ischemic brain damage by increasing p97-mediated surface GluA1-containing AMPA receptors
Source: J Transl Med. 2023 Aug 24;21:567. doi: 10.1186/s12967-023-04452-5 (PMC10463885; doi:10.1186/s12967-023-04452-5)
Supplement: Supplementary file 1 — Additional file 1: Figure S1. The NLRP3−/− and caspase-1−/− mice were genotyped by PCR with reverse transcriptase using mouse tail-tip DNA and mixed primers. The protein levels of NLRP3 and caspase-1 were assessed by Western blot in the brain tissues from WT, NLRP3−/− and caspase-1−/− mice. a The results of PCR showed a band of 666 bp in WT mice, and a band of 850 bp in NLRP3−/− mice. The results of Western blot showed no NLRP3 expression in NLRP3−/− mice. b The results of PCR showed a band of 500 bp in WT mice, and a band of 300 bp in caspase-1−/− mice. The results of Western blot showed no caspase-1 and clv-caspase-1 expression in caspase-1−/− mice. Figure S2. Co-IP assays show no interaction between GluA2 and p97 in the primarily cultured neurons with or without OGD treatment. Figure S3. TTC staining shows an improvement of infarction caused by HI with the treatment of AC-YVAD-CMK. a Representative TTC staining coronal brain sections (2 mm). Sections are labeled as five different levels (level 1–level 5) along the anterior (A) to posterior (P) axis. b Quantification of the cerebral infarct area in brain sections (infarcted area ratio (%) = white infarcted area/brain slice area). [file 12967_2023_4452_MOESM1_ESM.doc]

**Additional figures and figure legends**

**
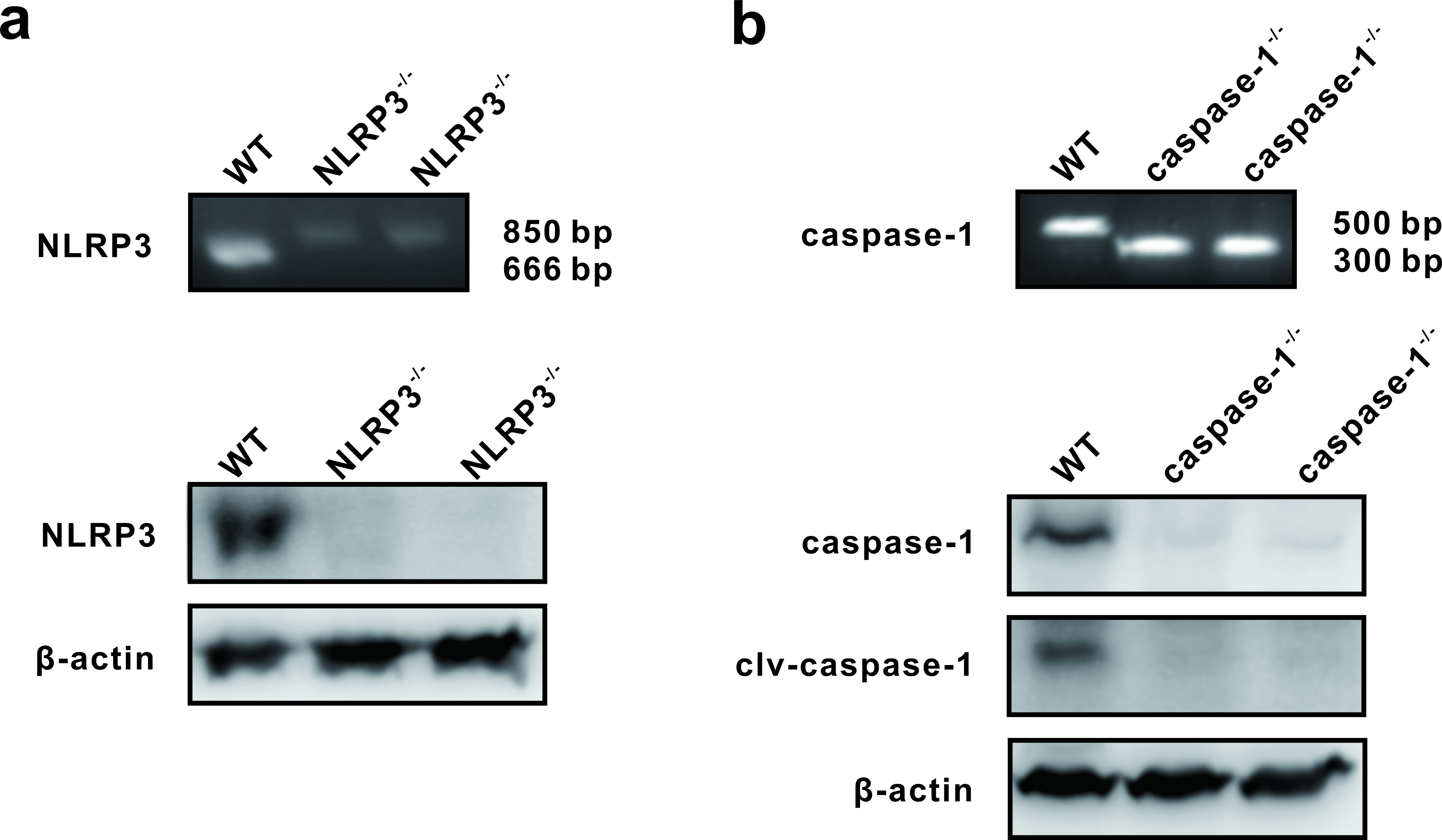
**

**Fig. S1** The NLRP3^-/-^ and caspase-1^-/-^ mice were genotyped by PCR with reverse transcriptase using mouse tail-tip DNA and mixed primers. The protein levels of NLRP3 and caspase-1 were assessed by Western blot in the brain tissues from WT, NLRP3^-/-^ and caspase-1^-/-^ mice. **(a)** The results of PCR showed a band of 666 bp in WT mice, and a band of 850 bp in NLRP3^-/-^ mice. The results of Western blot showed no NLRP3 expression in NLRP3^-/-^mice. **(b)** The results of PCR showed a band of 500 bp in WT mice, and a band of 300 bp in caspase-1^-/-^ mice. The results of Western blot showed no caspase-1 and clv-caspase-1 expression in caspase-1^-/-^mice.

**
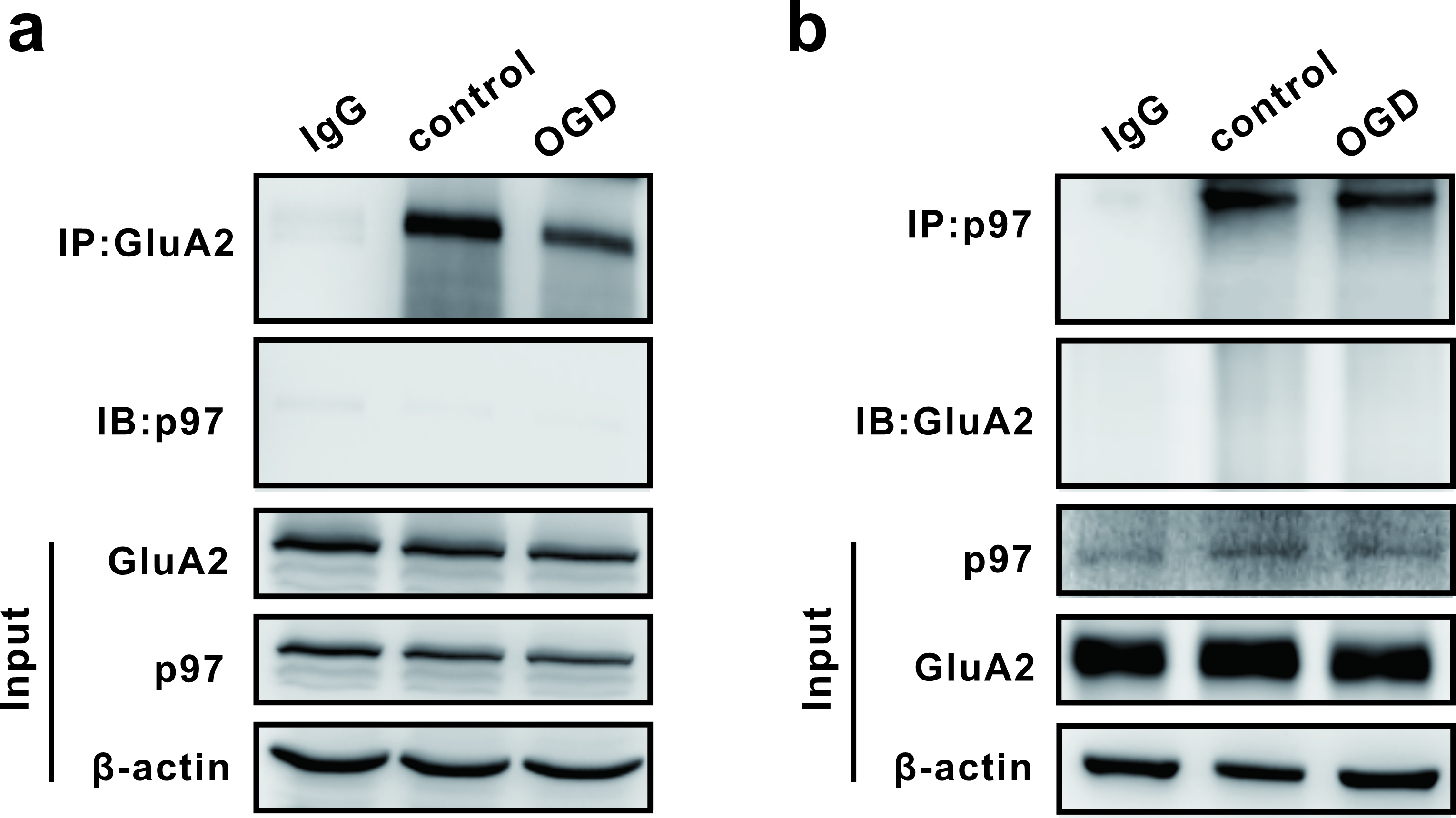
**

**Fig. S2** Co-IP assays show no interaction between GluA2 and p97 in the primarily cultured neurons with or without OGD treatment.


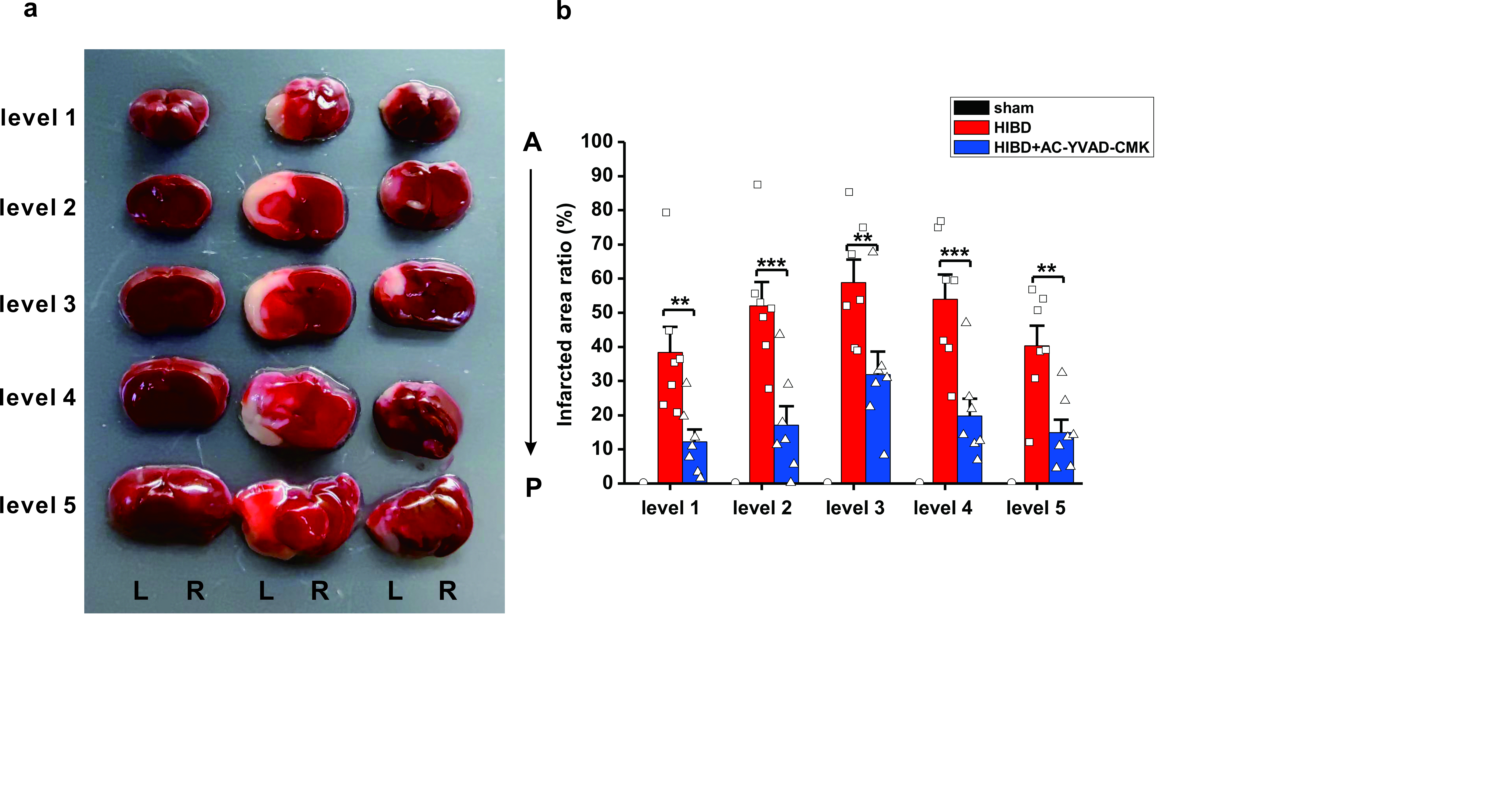


**Fig. S3** TTC staining shows an improvement of infarction caused by HI with the treatment of AC-YVAD-CMK. **(a)** Representative TTC staining coronal brain sections (2 mm). Sections are labeled as five different levels (level 1–level 5) along the anterior (A) to posterior (P) axis. **(b)** Quantification of the cerebral infarct area in brain sections (infarcted area ratio (%) = white infarcted area/ brain slice area).
